# Role of early shoulder tomography on the obstetric brachial plexus palsy

**DOI:** 10.1590/1413-78522015230101023

**Published:** 2015

**Authors:** Bruno Liberato de Souza Silva, Luiz Koiti Kimura, Bruno Eiras Crepaldi, Rames Mattar, Álvaro Baik Cho, Rubén Montiel Oviedo

**Affiliations:** Universidade de São Paulo, Faculdade de Medicina, Hospital das Clínicas, São Paulo, SP, Brazil, Instituto de Ortopedia e Traumatologia do Hospital das Clínicas da Faculdade de Medicina da Universidade de São Paulo, São Paulo, SP, Brazil

## Abstract

**Objective:**

To demonstrate the importance of performing early shoulder tomography in patients with obstetric brachial plexus palsy (OBPP).

**Methods:**

This series of cases retrospective study with level evidence IV was conducted by consulting 76 patient's medical records with OBPP divided into three age groups: ≤12 months, 13 to 24 months and ≥ 25 months. The patients were classified according to gender, affected side, type of paralysis according to Narakas classification, and by computed tomography, according to the Waters scale.

**Results:**

The association between the age groups with Waters classification was statistically significant (p=0,006), showing that patients in the group aged less than 12 months and in the group aged between 12 and 24 months had a relevant correlation between the physical examination and Waters > III when compared to patients from groups aged 25 months or older.

**Conclusion:**

This study shows a correlation between the findings in the physical examination and severe dysplasia on the shoulder of children under 24 months of age, justifying the early tomographic shoulder exam in order to achieve a better follow-up and a consider a more aggressive approach in the treatment of OBPP affected children. Level of Evidence IV, Case Series.

## Introduction

The obstetric brachial plexus palsy (OBPP) is a flaccid paralysis upper limbs caused by the exerted traction during childbirth maneuvers, usually due to dystocia of the scapular waist.^1^ The brachial plexus injury is caused by the stretching of the nerve trunks or root avulsion may be complete, with injuries in all roots. The high paralysis, also known as Erb-Duchene proximal paralysis, is more common and affects the C5-C6 roots. The impairment of the lower trunk from C8 to T1 causes hand paralysis, named Klumpke lower paralysis.

Most OBPP patients recover spontaneously in the early months of life.^2^^-^ 4 However, in some situations, muscle imbalance is observed due to the absence of abduction and external rotation which, over time, evolves to internal rotation contracture of the shoulder.^5^ This condition brings significant limitations in activities of daily living, and in the medium/long term it can evolve into posterior shoulder subluxation and change of the humeral head and the glenoid cavity.^6^^,^ 7

In order to show the evolution of the glenohumeral joint deformities, Bae *et al.* 5 developed a classification that takes into account the changes in joint congruence and deformities in the humeral head and glenoid, evaluated by axial computed tomography (CT) , similarly of the changes observed in the hip dysplasia.

A variety of surgical procedures have been proposed throughout history in order to improve the function of the upper limb and preventing the glenohumeral changes. They include exploration of the brachial plexus, conventional grafting, accessory nerve neuroticism, arthroscopic or open release of the internal rotation contracture of the shoulder, stretching of internal rotators, tendon transfers, open reduction of the glenohumeral joint and derotational osteotomies.^8^^-^ 13

Paradoxically, a higher incidence of patients with obstetrical paralysis below 24 months with severe shoulder dysplasia (Waters greater than or equal to IV) has been observed in our service. In the current literature there is no consensus on the criteria for the time indication of the performance of imaging studies to evaluate the deformities of the humeral head and the glenoid cavity.^14^^,^ 15

This study aims to discuss the importance of early carry out of shoulder tomography in patients with OBPP.

## Cases and methods

The observational, descriptive and retrospective study of case series, with level of evidence IV was submitted and approved by the Research Ethics Committee, in order to comply with Resolution No. 196/96 of *Conselho Nacional de Saúde*. The study was performed through consultation of records of 76 patients with OBPP at the Hand and Microsurgery outpatient service at *Instituto de Ortopedia e Traumatologia, da Faculdade de Medicina da Universidade de São Paulo,* São Paulo, SP, Brazil.

**Figure 1 A-B.**
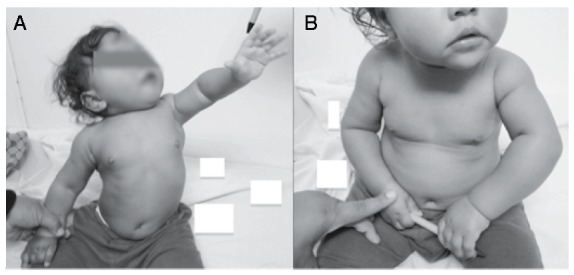
Clinical chart showing the presence of asymmetric skin folds in the axillary area, relative shortening of the left upper limb and the progressive loss of external rotation.

The research included OBPP patients who underwent Computed Tomography (CT) for classification according to the Waters and Peljovich scale,^10^ requested when they showed at the physical examination: presence of asymmetric skin folds at the axillary area, relative shortening of the affected upper limb and progressive loss of external rotation with the persistent adducted limb, as shown in Figure 1A-B.^10^^-^ 14

This study excluded patients who had inadequate medical records or abandoned follow up.

**Chart 1.**
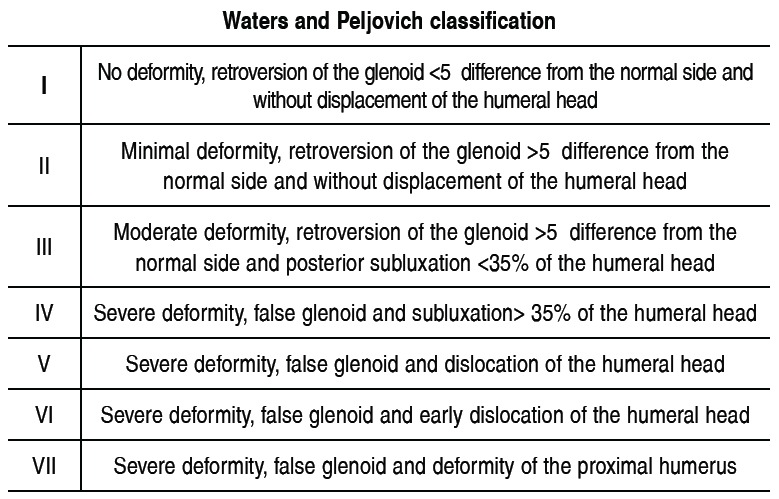
Waters and Peljovich classification of glenohumeral deformities caused by OBPP.

Patients were divided into three groups according to age at diagnosis at CT exam: group I) patients aged ≤ 12 months, group II) patients aged 13-24 months and group III) patients aged> 25 months. Patients were further classified according to gender, affected side and type of paralysis. According to Narakas,8 they were classified into the following types: type 1 - involvement of C5 and C6; type 2 - involvement of C5, C6 and C7; type 3 - total paralysis without Horner signal and type 4 - total paralysis with Horner signal. Patients were also classified using the Waters and Peljovich10 scale for glenohumeral deformities which assesses joint congruity of the proximal humerus and its correlation with the glenoid cavity. (Tables 1-2, Chart 1).

**Table 1.** Division of groups by gender and affected side.

Groups		< 12 months	12 to 24 months	> 24 months	Total (n)
Gender	Masculine	6(46%)	5(18%)	30(66%)	41
	Feminine	7(54%)	13(82%)	15(34%)	35
Total (n)		13	18	45	76
Side	Right	8(62%)	12(67%)	23(51%)	43
	Left	5(38%)	6(33%)	22(49%)	33
Total (n)		13	18	45	76

**Tabela 2.** Division of groups regarding the type of paralysis according to Narakas classification.

Groups		< 12 months	12 to 24 months	> 24 months	Total (n)
Narakas (n%)	Type I	8(24%)	11(32%)	15(44%)	34
	Type II	3(14%)	3(14%)	16(72%)	22
	Type III	2(11%)	3(16%)	14(73%)	19
	Type IV	0	1(100%)	0	1
	Total (n)	13	18	45	76

Patients were considered with mild glenohumeral change those with score levels I, II and III according to Waters classification and severe glenohumeral change those with imaging test scores levels IV- VII. Imaging studies were evaluated by two experienced surgeons and, in case of disagreement, a third reviewer was consulted for the final characterization of the score.

The collected data were cataloged in Microsoft Excel. Statistical analysis were performed using ANOVA and post hoc for the correlation between the Waters scale and age groups, and the Kruskal-Wallis test to assess Narakas classification. Values of p were considered statistically significant when <0 05.

## Results

**Figure 2.**
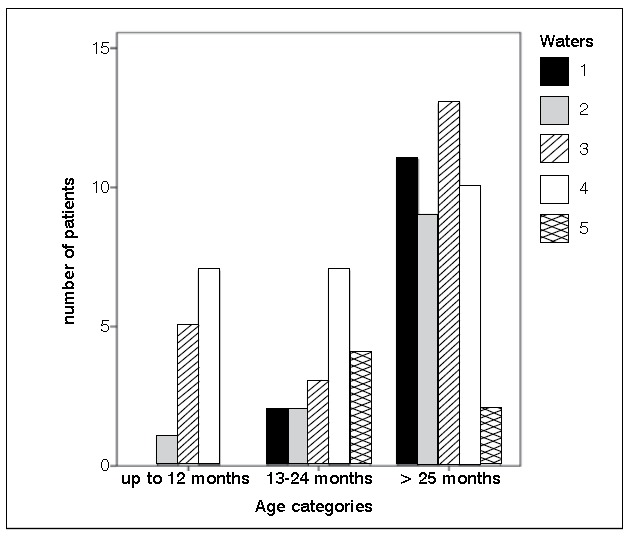
Categorization of patients according to Waters and Peljovich scale.

Regarding the categorization of patients according to Waters and Peljovich10 classification, we can observe their distribution between the groups in Figure 2.

**Figure 3.**
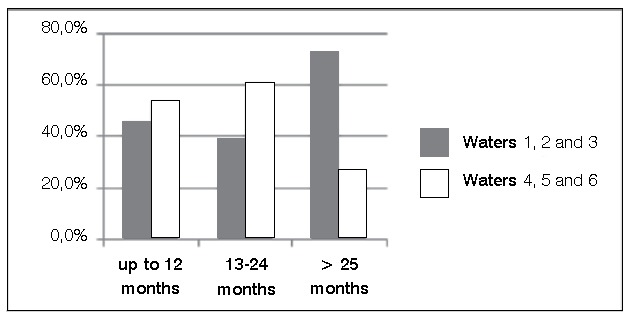
Categorization of patients according to Waters and Peljovich seriousness scale. Fonte: http://danielrestituyo.blogspot.com.br.

When we categorize the shoulder alterations as mild (Waters I, II and III) or severe (Waters IV, V and VI), we observed the following behavior of the groups shown in Figure 3.

**Figure 4.**
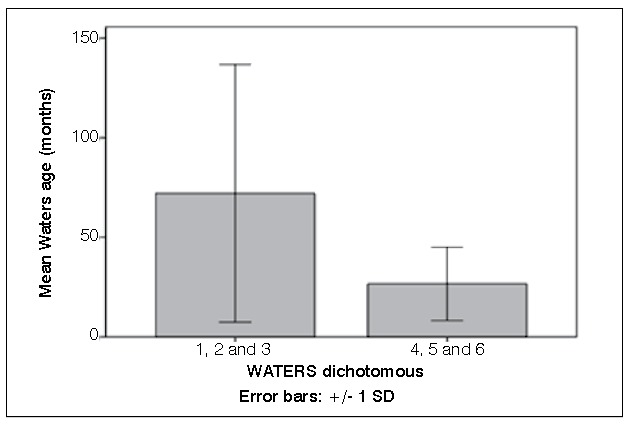
Age correlation with Waters scale divided into slight change (1,2 and 3) and serious deterioration (4, 5 and 6).

The correlation between the three groups of patients and the type of paralysis presented was not statistically significant (p=0.117). Regarding the comparison to Waters classification, statistical analysis showed statistical significance between the groups, however, because there are three groups we cannot infer which of them was significant. Upon this fact, comparisons were made by grouping two stages. Firstly, we compare groups 1 and 2 with group 3, and the result was statistically significant (p = 0.006). Secondly, we compared group 1 with groups 2 and 3, not being statistically significant. We can say with these data that there is a significant relationship between afore mentioned physical exam with the serious deterioration of the glenoid (Waters IV, V and VI), in groups 1 and 2. (Figure 4)

## Discussion

At the beginning of last century the glenohumeral posterior dislocations in children were often reported.^7^^,^ 16 ^,^ 17 The evaluation of these patients, at that time, was performed clinically and radiology was not routinely used, since other imaging tests such as ultrasound and CT scan did not exist then.

Whitman^16^ was the first to coin the term "congenital dislocation" and set out its three variants. The first variant was actually the dislocation *in uterus* of the joint. The second was a result of trauma at birth, derived mainly from fetal extraction maneuvers. The third and most common results of muscle imbalance generated by paralysis of muscle groups.

Fairbank^7^ in one of his series described 28 cases of posterior glenohumeral dislocation in 37 children evaluated, pointing as a possible cause of this deformity the asymmetry of muscular strength generated by the obstetric paralysis. Since the first descriptions only nine cases of posterior dislocation in children in the first year of life had been reportados.^18^^,^ 19 Troum *et al.* 18 stated that: "it is difficult to diagnose severe glenohumeral change in children under one year of age, therefore, the condition is rare and can often go unnoticed". One of the difficulties of this pathology lies on the diagnosis at the right time, which could improve the functional prognosis. 

**Figure 5.**
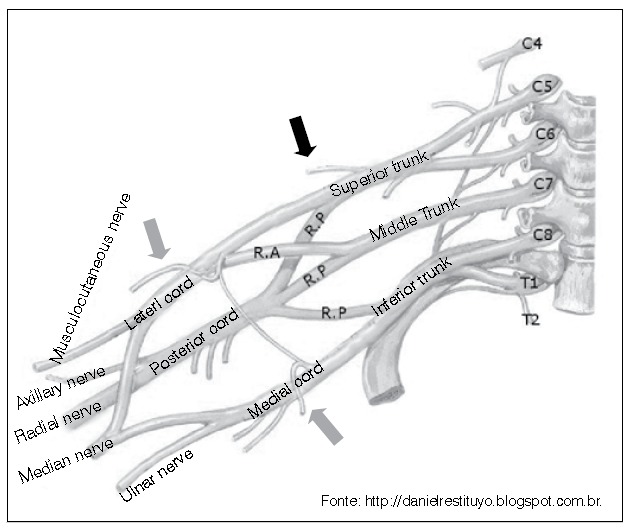
Brachial Plexus. Black arrow: suprascapular nerve. Green arrows: medial and lateral pectoral nerves.

These changes secondary to neurological injuries are due to impairment of the nerves responsible for external rotation (suprascapular nerve) and the preservation of the nerves responsible for the innervation of the internal rotators (medial and lateral pectoral nerves). (Figure 5)

Currently, we can evaluate children through physical examination, being suggestive of serious change: asymmetric skin folds in the axillary area, relative shortening of the affected upper limb and the progressive loss of external rotation with the limb adducted.

Children under 24 months have flaccid limb paralysis, which gradually evolves into posture in internal rotation, due to lack of external rotators and adequate rehabilitation, associated to the fact that the internal rotators present shortening, leading to adducted posture and internal rotation of the shoulder. Therefore, when we observe important limitation of external rotation of the affected limb, associated with apparent shortening and asymmetric folds in the axillary region in children under 24 months, the probability of serious shoulder alteration is greater, as shown by our study. (Figures 3 and 4) In the presence of any of these signs, particularly the loss of external rotation, imaging studies such as Computed Tomography is indicated.^14^

The most common pattern of brachial plexus injury associated with severe glenohumeral change was Narakas^8^ type 1 for groups aged ≤ 12 months and 13-24 months. As exposed above this is due to lack of external rotators function compromised by the upper trunk injury, while the internal rotators are preserved by the integrity of the posterior and medial cord.

The oval shape of the humeral head, now in external rotation, presses the back of the glenoid, causing a flattening that progressively increases and results in further displacement of the humerus.

However, In the group of children aged ≥ 25 months the difference between Narakas' types 1 and 2 was not statistically significant, which is consistent with the literatura.^14^^,^ 15 In this group we must consider that the patients underwent surgical procedures, both neurological and tendon releases and muscle stretching influencing the functional prognosis and possibly the anatomical changes in the glenohumeral joint.

This fact may explain the statistical differences observed between the groups aged < 24 months and >25 months regarding correlation with physical examination and CT imaging. Upon these findings, there is need for a new study detailing this difference and its correlation with performed surgical procedures in the evolution of these children.

We observed a difficulty in early diagnosing, which can be corroborated by the high mean age of the first imaging study performed in our service at 14 months, compared to six months on average found by Moukoko *et al.*,^14^ in Dallas, USA. This fact somehow can reflect the difficulty of monitoring these patients in the major centers of developing countries.

We found no data in the literature regarding the standardization of imaging requests to assess the glenohumeral deformity, however, the current study points to a statistically significant correlation between the physical findings mentioned and serious shoulder alteration in children under 24 months of age, justifying these imaging studies, which may provide better monitoring and early treatment of children with OBPP.

This study presents as limitation not taking into account the previous surgeries to imaging studies performed in patients.

We believe in keeping this line of research looking at a larger study group, as a cohort, taking into account both the clinical, physical examination, imaging and surgeries to improve and create a common protocol for the follow-up of this disease.

## Conclusion

This study showed a statistically significant correlation between the physical findings and serious changes in CT of children's shoulder of groups aged ≤ 12 months and aged 13-24 months, justifying the early performance of imaging examination, aimed at better monitoring and more aggressive approach in the treatment of children with OBPP.
